# Exploring natural breast symmetry in the female plastic surgical patient population

**DOI:** 10.3205/iprs000173

**Published:** 2023-07-07

**Authors:** Helga Henseler

**Affiliations:** 1 Klinik am Rhein, Klinik für Plastische und Ästhetische Chirurgie, Düsseldorf, Germany

**Keywords:** breast dimensions, linear distances, anthropometry, symmetry, shape, three-dimensional imaging, Vectra, objective analysis

## Abstract

**Background:**

Breast symmetry remains a challenging quality to measure. The question arises of how much baseline breast symmetry exists in the female plastic surgical patient population.

**Material and methods:**

Several linear dimensional assessments were collected based on a retrospective analysis of objective data of women with natural breasts, who presented for an initial consultation in a plastic surgical clinic and were measured with the 3D Vectra Camera by the company Canfield Scientific Inc., U.S.A. The first 100 cases were extracted from the large database in alphabetical order. The nipple positions were examined, including the following linear dimensions: distances from the jugulum to the nipple, from the nipple to the inframammary fold, and from the nipple to midline. Furthermore, the under-breast widths were obtained.

**Results:**

The objective three-dimensional imaging method determined that all patients had some degree of asymmetry. The linear distances from the jugulum and midline to the nipple revealed that the measurements were larger on the left side. However, the nipple to the inframammary fold measurements were roughly the same. In the sample of plastic surgical patients, the average distance between the jugulum and nipple was around 20 cm, the distance between the nipple and inframammary fold was around 6.5 cm, the distance between the nipple and midline was around 10 cm, and the under-breast width was around 13 cm. On average, the jugulum-nipple distance on the right side was 19.9±2.3 cm and 20.1±2.4 cm on the left side. The distance between the nipple and the inframammary fold was 6.4±1.1 cm, revealing a similar distribution on both sides. The mean distance from the nipple to the midline was much larger on the left side of the body at 10.0±1.2 cm than on the right side at 9.4±1.3 cm. The under-breast width on the right side was slightly larger than the left side, with measurements of 13.4±1.8 cm versus 13.2±1.7 cm.

**Conclusion:**

Breast dimensions can be described comprehensively by an objective three-dimensional imaging process, which can detect asymmetry in all patients. The differences related to the nipple position were larger on the left side than the right regarding the distances from the jugulum and particularly the midline to the nipple, which seems to be of special importance, but not from the nipple to the inframammary fold. The linear measurements for the distances from the jugulum and the midline to the nipple are essential to accurate symmetry assessments and form an aesthetic triangle of the breast, which is a new term and the key to a simplified shape analysis.

## Zusammenfassung

**Hintergrund:**

Brustsymmetrie bleibt schwierig zu messen. Die Frage stellt sich, wie viel grundlegende Brustsymmetrie in der weiblichen, plastisch-chirurgischen Patientenpopulation existiert.

**Material und Methode:**

Basierend auf einer retrospektiven Analyse von objektiven Daten von Frauen mit natürlichen Brüsten, die sich für eine initiale Konsultation in einer plastisch-chirurgischen Klinik vorstellten und die mittels der 3D-Vectra-Kamera der Firma Canfield Scientific Inc., U.S.A., vermessen wurden, wurde eine Reihe von Messdaten linearer Dimensionen gesammelt. Die ersten 100 Fälle wurden aus der umfangreichen Datenbank in alphabetischer Ordnung entnommen. Die Positionen der Brustwarzen wurden unter Einschluss folgender linearer Dimensionen untersucht: die Distanzen vom Jugulum zur Brustwarze, von der Brustwarze zur Unterbrustfalte und von der Brustwarze zur Mittellinie. Außerdem wurden die Brustbasisbreiten festgehalten.

**Ergebnisse:**

Die dreidimensionale Bildaufnahmemethode ergab, dass alle Patientinnen einen gewissen Grad an Asymmetrie aufwiesen. Die linearen Abstände vom Jugulum und der Mittellinie zur Brustwarze ergaben, dass die Messwerte auf der linken Seite größer waren. Demgegenüber waren die Messwerte von den Brustwarzen zu den Unterbrustfalten ungefähr ähnlich. In der Gruppe der plastisch-chirurgischen Patientinnen betrug die durchschnittliche Distanz zwischen dem Jugulum und der Brustwarze ca. 20 cm, die Distanz zwischen der Brustwarze und der Unterbrustfalte ca. 6,5 cm, die Distanz zwischen der Brustwarze und der Mittellinie ca. 10 cm, und die Brustbasisbreite ca. 13 cm. Die Distanz zwischen dem Jugulum und der Brustwarze auf der rechten Seite betrug im Durchschnitt 19,9±2,3 cm und 20,1±2,4 cm auf der linken Seite. Die Distanz zwischen der Brustwarze und der Unterbrustfalte betrug 6,4±1,1 cm, mit einer ähnlichen Verteilung auf beiden Seiten. Die mittlere Distanz zwischen der Brustwarze zur Mittellinie ergab sich als deutlich größer auf der linken Seite des Körpers mit 10,0±1,2 cm als auf der rechten Seite mit 9,4±1,3 cm. Die Unterbrustbasisbreite war auf der rechten Seite geringfügig größer als links mit Messungen von 13,4±1,8 cm und 13,2±1,7 cm.

**Schlussfolgerung:**

Brustdimensionen können umfassend durch einen objektiven dreidimensionalen Bildaufnahmeprozess beschrieben werden, der Asymmetrie in allen Patientinnen aufdecken kann. Im Hinblick auf die Brustwarzenposition waren die Messdaten größer auf der linken Seite als auf der rechten in Bezug auf die Distanzen vom Jugulum und besonders von der Mittellinie zur Brustwarze, was von besonderer Bedeutung zu sein scheint, aber nicht von der Brustwarze zur Unterbrustfalte. Die linearen Messungen der Abstände vom Jugulum und der Mittellinie zur Brustwarze sind essentiell, um Symmetrieuntersuchungen durchzuführen, und formen ein ästhetisches Dreieck der Brust, welches ein neuer Terminus ist und den Schlüssel zu einer vereinfachten Formanalyse darstellt.

## Introduction

The human breast presents itself in multiple shapes and sizes. Traditionally, the breast was judged mainly by subjective expert opinion [1]. However, this method fails to alleviate the challenge of comprehending the complexity of breast dimensions. Multiple modes of investigation, measurements, and judgments have been undertaken [2].

To date, a two-dimensional breast assessment approach using an anthropometric method with a simple tape measure is standard [3]. Due to the simplicity of this method and the low cost and ready-to-use availability, the application of tape measurements is widespread in plastic surgery clinics worldwide. The two-dimensional anthropometric approach is a significant advancement from a mere subjective system in breast assessment.

The developments of the digital age and improvements in software calculations have provided technical support for doctors beyond a merely subjective or anthropometric approach in recent times. In the early years, only a few digital prototype systems were available, and the progress of the implementation into medical use was slow [4]. Objective assessment tools, such as the three-dimensional imaging methods, have mainly evolved to be helpful in the last two decades.

Discussions concerning the medical usage of objective imaging methods became more frequent. Possible applications for diagnostic or therapeutic indications were evaluated. Questions emerged regarding the potential role of three-dimensional digital photography in breast surgery [5]. For a long time, three-dimensional imaging did not have sufficient power to help improve surgical results. Nevertheless, research was pursued in this field, which slowly developed.

Despite the technical advances, the subjective assessment and anthropometric measurement methods continued to be applied during daily clinical activities and research investigations [6]. The high costs for professional imaging systems partly prevented broader usage. In clinical practice, the amount of breast and chest wall symmetry found in a patient group that presented for breast augmentation was considered interesting [7]. The analysis was based retrospectively on four examiners and their subjective judgment of patient photographs. They found that chest wall asymmetry plays a vital role in breast augmentation and that 88% of the examined women had some degree of asymmetry.

In line with this study, the role of three-dimensional imaging as a helping tool in cases of breast assessments was questioned [8]. It was postulated that a correction of breast asymmetry would not exist. The article outlined how, even after surgical interventions, a certain degree of asymmetry would always exist. Following this publication, a discussion emerged about the role of three-dimensional imaging in breast surgery, especially regarding corrective surgery in cases of asymmetry.

While objective methods provide the option to detect even minimal degrees of breast asymmetry, the following question arises: what is the baseline breast symmetry in the general female plastic surgical patient population that presents itself in the clinic for a consultation?

## Objectives of this study

It was interesting to determine the degree of baseline breast symmetry in a female plastic surgical patient population. The objective analysis of naturally occurring breast dimensions based on the modern three-dimensional imaging method was assessed.

## Material and methods

**Figure 1:**
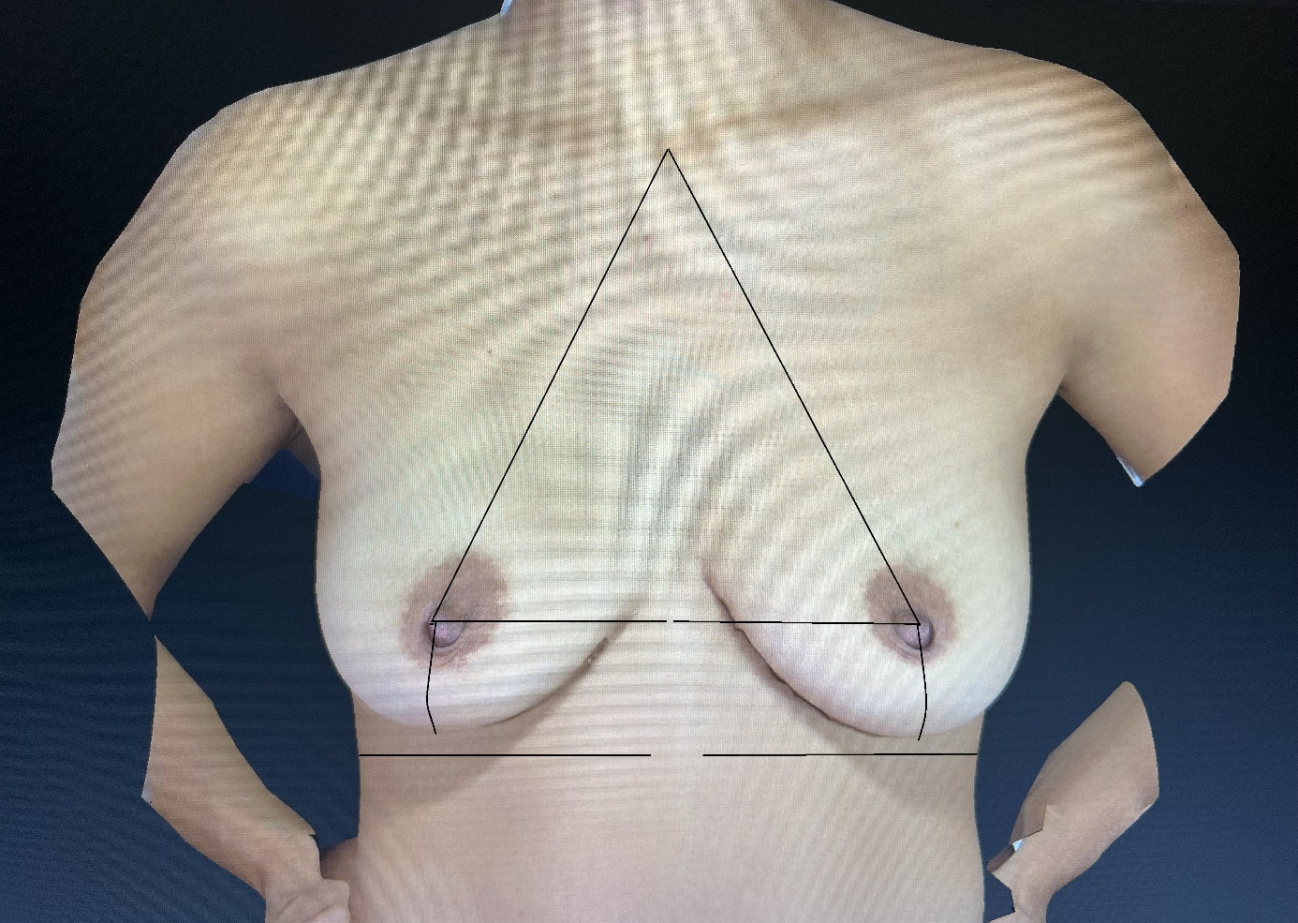
The four linear distances of a female breast of interest

Symmetry data were extracted from the extensive database of a plastic surgery clinic. Based on a retrospective analysis of the natural breasts of women who presented for an initial consultation and were captured with the 3D Vectra Camera by the company Canfield Scientific Inc., U.S.A., several linear dimensional assessments were collected. The women who presented for the initial consultation were asked on admission about the purpose of the visit. Those women who indicated possible breast implant surgery, either in the majority for augmentation or in the minority for asymmetry correction purposes, were captured with the 3D Vectra camera via the front desk staff ahead of the consultation with the plastic surgical consultant. Through this procedure 3D breast images were readily available for the consultation, and the initial breast analysis as well as possible suitable simulations could be conducted. The first 100 cases were extracted from the database in alphabetical order. The inclusion criteria were women with natural breasts without previous surgery; the exclusion criteria were postoperative women and those with poor imaging results. The nipple positions were examined, and the following linear breast dimensions were obtained: from the jugulum – also known as the sternal notch – to the nipple, the nipple to the inframammary fold – also known as the submammary or lower breast fold, the nipple to the midline, and, further, the under-breast width (Figure 1 (Fig. 1)).

Breast symmetry was investigated, and statistical analysis was conducted with the program STATISTICA by StatSoft, Inc., U.S.A.

## Results

The objective three-dimensional imaging method of this study determined that all patients had some degree of asymmetry. The linear distances from the jugulum and midline to the nipple revealed that the measurements were larger on the left side. However, the nipple to the inframammary fold measurements were roughly the same.

In the sample of plastic surgical patients, the average distance between the jugulum and nipple was around 20 cm, the distance between the nipple and inframammary fold was around 6.5 cm, the distance between the nipple and midline was around 10 cm, and the under-breast width was around 13 cm. 

### Jugulum to nipple distances

The average jugulum-nipple distance was 19.9±2.3 cm on the right side, and 20.1±2.4 cm on the left side. The mean absolute difference of these measurements was 0.67±0.56 cm or 3.3±2.8%. Additionally, the maximal deviation was 2.7 cm or 14.4% (Table 1 (Tab. 1)).

**Figure 2:**
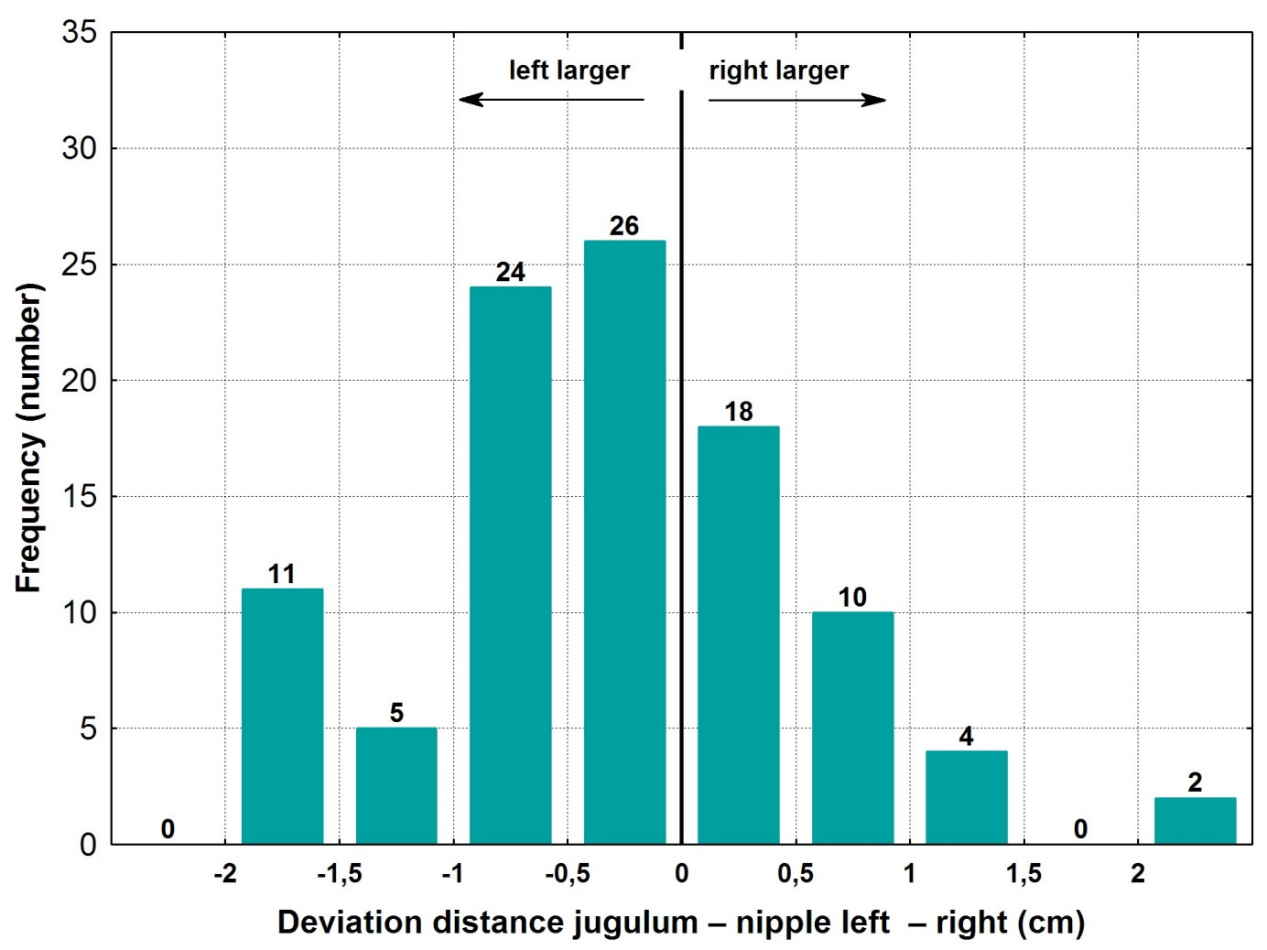
Deviation of left versus right jugulum to nipple distances

Figure 2 (Fig. 2) displays the distribution of the frequency of the deviations between the right and left breasts. Altogether, 44 of the 100 cases were within ±0.5 cm deviance. Larger deviances of more than ±2 cm were seen rarely, in only 2 cases.

### Nipple to inframammary fold distances

**Figure 3:**
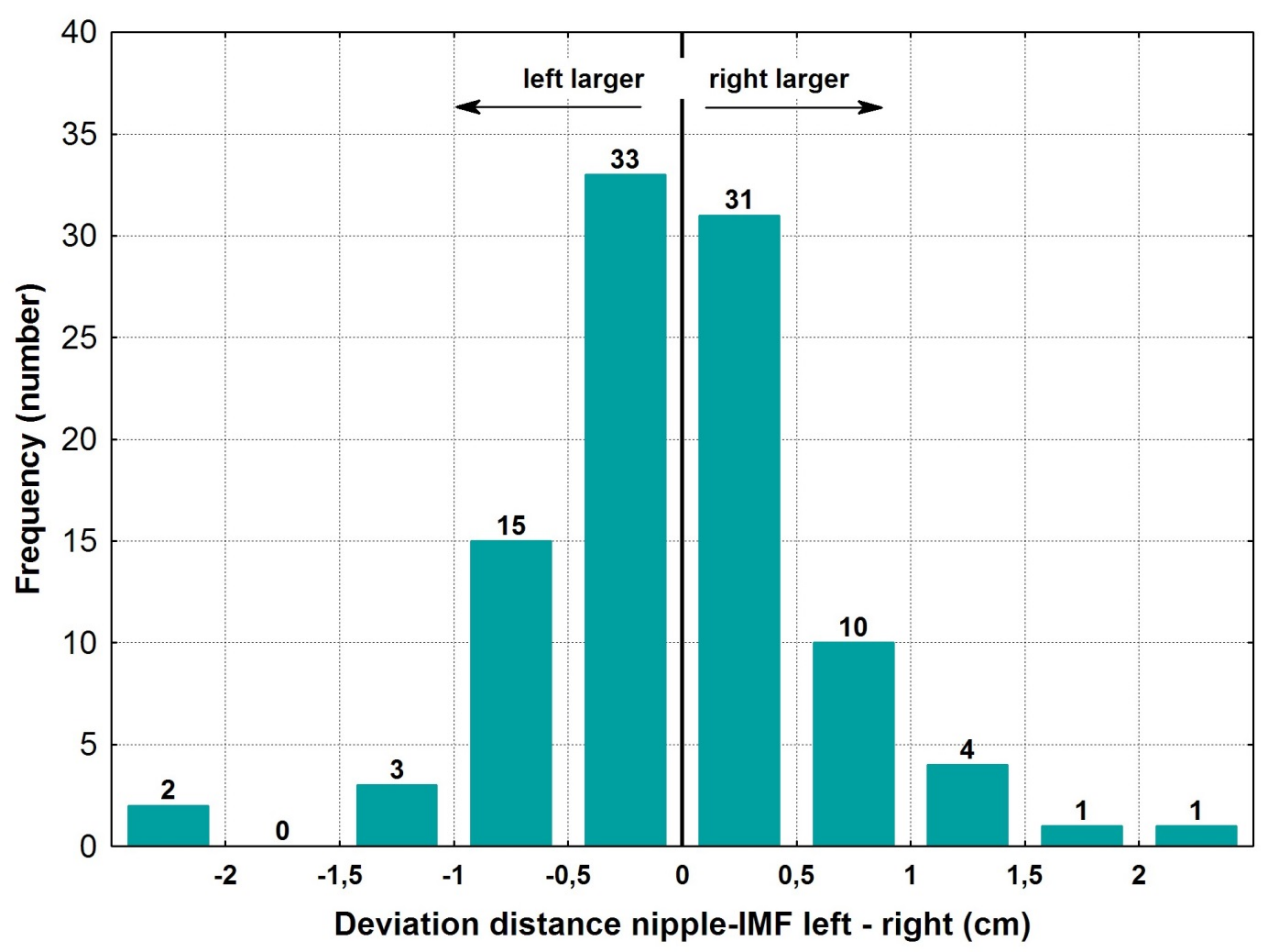
Deviation of left versus right nipple to inframammary fold (IMF) distance

The mean distance between the nipple and the inframammary fold was 6.4±1.1 cm, with a mean absolute difference of 0.53±0.35 cm or 8.1±5.5% (Table 2 (Tab. 2)). The variations between the right and left sides revealed a relatively symmetrical distribution, as seen in Figure 3 (Fig. 3). 64 out of 100 cases were positioned within ±0.5 cm of deviation. Significant variations of more than ±1 cm were only found in 11 out of 100 patients.

According to the statistical analysis, 69% of the absolute deviations were within ±10% difference between the right and the left side of the body.

Given the nipple to inframammary fold distance, 64% of cases were within 0.5 cm, and deviations of more than 2 cm were found in 3 cases.

### Nipple to midline distances

**Figure 4:**
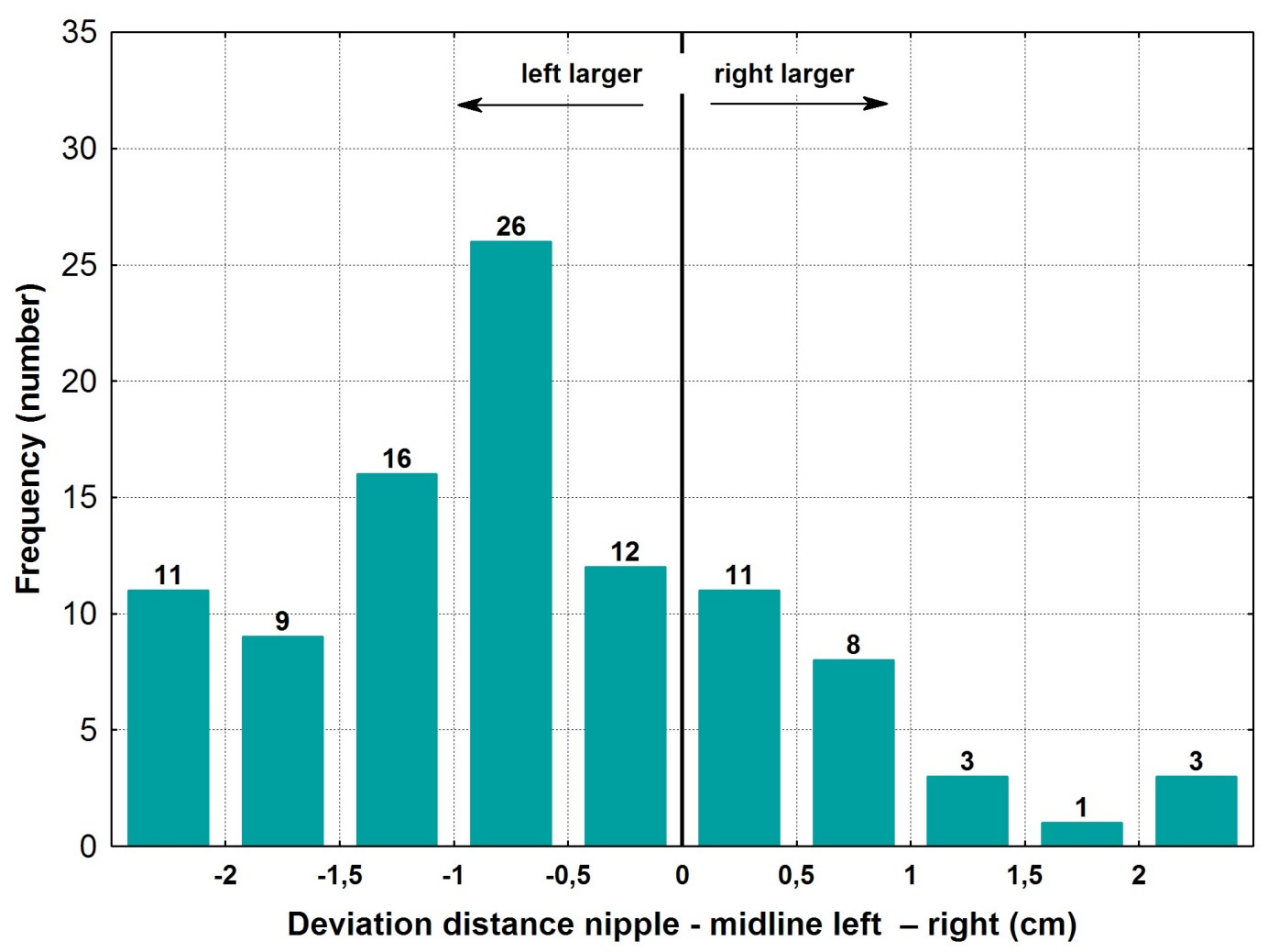
Deviation of left versus right nipple to midline distance

The mean distance from the nipple to the midline was much larger on the left side of the body at 10.0±1.2 cm, versus the right side at 9.4±1.3 cm. The mean absolute difference of both distances was 1.1±0.8 cm and 10.9±7.6% (Table 3 (Tab. 3)). Regarding the distance between nipple and midline, 23% were within a 0.5 cm deviation, and 14% presented with more than a 2 cm difference (Figure 4 (Fig. 4)).

In 27% of cases, there was a maximum absolute deviation between the nipple and the midline of 5%, and in 53% of cases the maximum was 10%.

### Under-breast width

The under-breast width was slightly larger on the right than on the left, with measurements of 13.4±1.8 cm versus 13.2±1.7 cm, respectively. The mean absolute difference of measurements from both sides was less than one centimeter, at 0.8±0.7cm (Table 4 (Tab. 4)). Regarding the under-breast width, 39% of cases were within a 0.5 cm deviation, and there were 10 cases with a variation of more than 2 cm.

**Figure 5:**
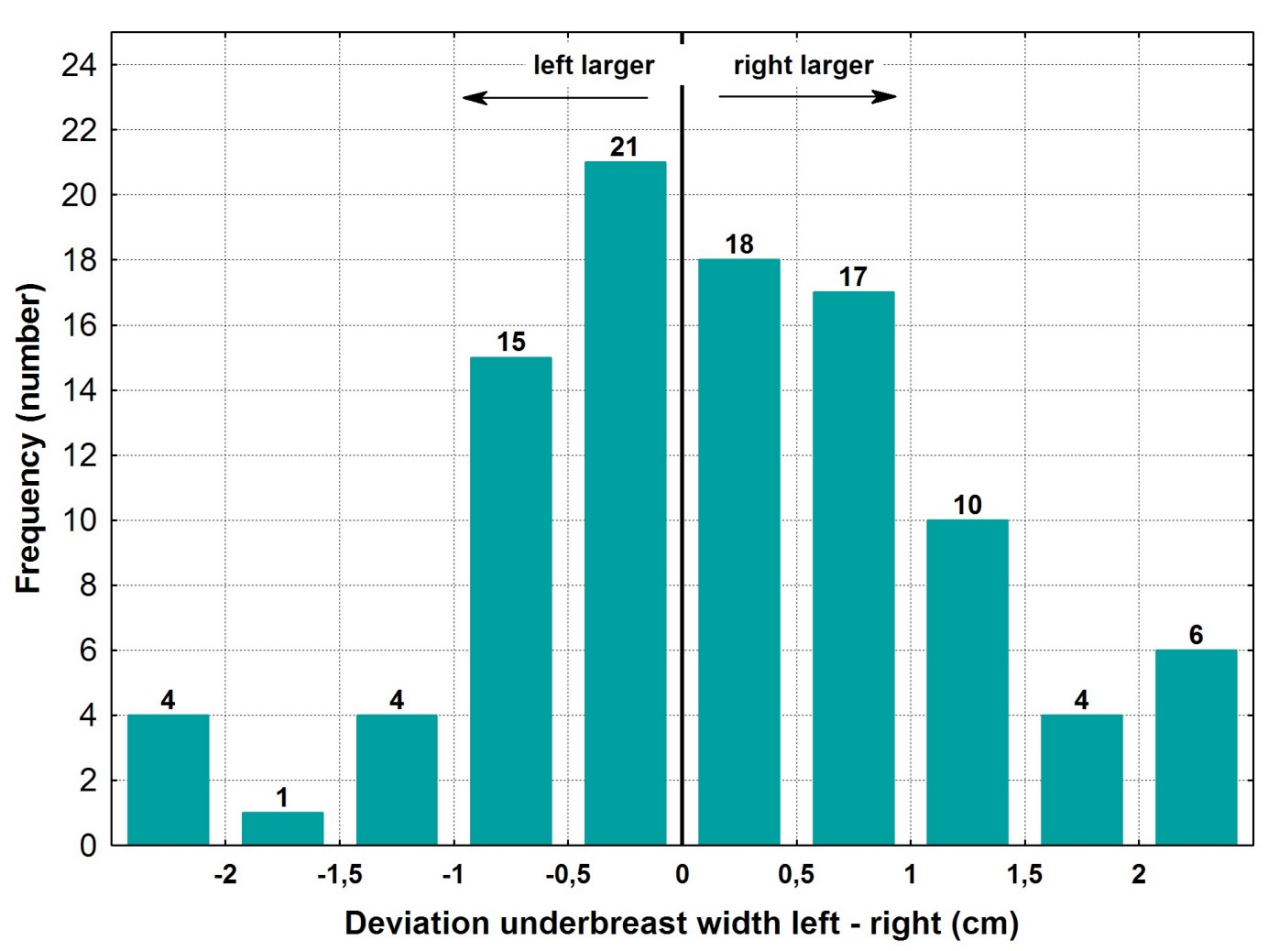
Deviation of left versus right under breast width

The distribution of the deviations between the right and the left side revealed a small number of cases in which the measurements were larger on the right side than on the left (55 versus 45 patients) (Figure 5 (Fig. 5)). In 83 out of 100 cases, the difference between the left and the right breast was less than 10%. In 51% of cases, there was a maximum absolute deviation of the under-breast width of 5%.

## Discussion

The patients investigated in this study were predominantly women with non-ptotic breasts due to the requirement of this study to include only complete images of good quality, which remains a problem in ptotic breasts. The three-dimensional capture system, the Vectra camera by Canfield Scientific Inc., was constructed to capture patients in an upright position. Therefore, patients with ptotic breasts show incomplete breast images in the inframammary fold. The lack of data in this area meant that only women with smaller breasts without a significant amount of breast ptosis could be included in this study.

Previous research has found a stability of breast volume assessments with the objective three-dimensional measurement method in contrast to the subjective method that presented larger estimation errors with increasing breast sizes [2], [9]. Therefore, while the study population presented here overall was one with smaller breasts, it could be postulated that data should also be valid for a group with larger breasts due to the usage of an objective modern three-dimensional measurement system. 

The previously reported problems of visualizing the ptotic breast shape [10] with the three-dimensional imaging method have continued to be challenging to overcome in due course despite the progress of the technique. Modern capture systems, such as the one by Canfield, are still predominantly applied for patients with smaller breasts who present for consultation regarding breast augmentation or mastopexy procedures. Alternatively, previously research was conducted with a prototype 3D imaging system to capture female breasts in a capture frame that supported women to bend forward to a nearly horizontal position of the undressed upper body, so that a complete image even of larger breasts could be obtained [4]. 

In comparison to breast volume assessments it further remains challenging to understand breast shapes based on various breast dimensions. Fortunately, these can be reduced to crucial measurements in a simplified manner. When looking at breast shape, several linear distances are used for description. The study presented here investigated four linear breast dimensions which were judged to be of crucial importance in breast surgery: jugulum to the nipple, nipple to the inframammary fold, nipple to midline, and under-breast width. However, breast shape needs to be differentiated from breast volume. In breast symmetry assessments, the shape is the feature under investigation as it is seen as a geometric figure [11]. Breast volume should be considered separately and was not a matter of this presentation. 

Linear distances help to assess breast symmetry as they can be obtained on either side. The relationship between breast symmetry and attractiveness has been investigated before. It was found that symmetry plays a role in the perception of attractiveness and that breast symmetry is a reliable signal of reproductive potential. Women with symmetric breasts were rated as more attractive and therefore were selected for mating more often, which could explain why they seem to have more children than others [12]. 

Despite these judgments, the high prevalence of breast asymmetry was previously outlined [8], [13]. Based on a single plastic surgical expert examination, breast asymmetry was detected in a retrospective review, and it was found that 83.3% of patients who complained postoperatively about asymmetry after breast augmentations had a constitutional breast or chest wall asymmetry. The author suggested educating patients to increase postoperative satisfaction [14]. 

In comparison, 100% of patients who underwent three-dimensional scanning showed breast asymmetry [15]. The incidence of asymmetry in women of 100% of the soft tissues of the breast or the chest wall was also confirmed in another study, which identified asymmetries of the breast or chest wall by 4D photography [16]. These findings were in line with the study group examined here. It seemed that the more precise the method of investigation was, the more breast and chest asymmetry was found. 

In spite of all the technical advances, subjective assessments continued to play a role in breast assessments. Based on subjective expert opinion, the role of chest wall deformities in breast augmentation was further investigated [17]. Various chest wall asymmetries were found, and the profiles of prostheses were discussed. However, the study was purely based on subjective observation, and no error assessment or objective measurement method was used. 

In daily clinical practice, plastic, aesthetic, and breast surgeons occasionally deal with patients who complain about their breast symmetry postoperatively. The unhappy patient remains a challenge. Patients aim for symmetrical breasts and are not easily satisfied if their desires are not met [14]. However, an essential question in this context would be how much natural breast symmetry exists in the population. Is the desire for perfection far from the reality of natural breast symmetry? One option to answer this question would be to pick a vast number of women randomly and to ask them to have their breasts photographed and analyzed. However, it was not feasible for the author to approach such a large group of women for this purpose, who would need to undress to capture and consent to the research method. This was why the method presented here was chosen: to resort to a retrospective study of data readily available from an extensive database of a plastic surgical clinic. As patients previously had presented themselves for plastic surgical consultation, they had consented and opted in their own interest to have their breasts imaged and analyzed, so that measurements for statistical analysis could be examined. As a possible limitation to the approach chosen in this study, it could be argued that this group of people might be different from the general population. However, the women studied here represent those that present themselves for consultation and, therefore, comprise the group of patients that surgeons have to deal with for procedure-planning purposes. 

As outlined, this study was based on four linear breast dimensions on either side to assess shape and symmetry in a simplified manner and stands in contrast to other attempts of research approaches that divide the breast into parts to find out about the ideal breast shape [18]. An observational study investigated the concepts of aesthetic breast dimensions regarding the ideal breast. Four key features of the ideal breast shape were published. The proportion of the upper to the lower pole, the angulation of the nipple upwards, the upper pole slope, and the convexity of the lower pole was found to be important. Further, a morphometric analysis of the perfect breast was conducted, investigating the views of the general public on ideal breast proportions [19]. When defining the ideal breast, a ratio of 45:55 of the upper to lower breast proportions was described. The same author investigated subjective observations and patient questionnaires to analyze breast shapes for planning breast augmentations [20]. The author felt to have provided a simple and reproducible formula for beautiful breast shapes. However, the four linear measurements, as presented in the study here, are predominantly used in daily clinical practice. The simplicity of the application seems to be a successful feature regarding its widespread use. The application of automated linear distance measurements by a three-dimensional imaging method introduced even further support for surgeons in comparison to obtaining those distances by application of a manual tape measure. 

With these developments, it is evident that various approaches, that is subjective as well as two and three-dimensional methods of breast analysis, will continue to be used. This is true for clinical assessments as well as for research studies. 

While there are many different ways to conduct a breast assessment, each method presents its indications, advantages, and disadvantages, and no common ground can be found for all aspects. While three-dimensional analysis seems to provide one of the most comprehensive approaches [21], tape measures are readily available, low cost, and valuable in breast assessment. As investigated here, a few key linear measurements are necessary for shape analysis in a simplified manner and are sufficient to report on breast symmetry. This study presented the new objective findings that linear measurements between the jugulum and the nipple tend to be larger on the left than on the right, which is particularly even more considerable regarding those from the nipple to the midline. In contrast, those measurements from the nipple to the lower breast fold are roughly the same between both sides. Not only could the study prove that in the natural breast the left side seems to be slightly larger, but it could also be postulated that the linear measurement for the distances of the nipple to the midline seems to be of special importance, which is a measurement that is not always applied in the daily clinical practice. The linear measurements for the jugulum and the midline to the nipple together form an aesthetic triangle of the breast, which is a new term and the key to a simplified shape analysis.

## Conclusion

Breast dimensions can be described comprehensively by an objective three-dimensional imaging process, which can detect asymmetry in all patients.

The differences related to the nipple position were more prominent on the left side than the right regarding the distances from the jugulum and particularly the midline to the nipple, which seems to be of special importance, but not from the nipple to the inframammary fold.

The linear measurements for the distances from the jugulum and the midline to the nipple are essential to accurate symmetry assessments and form an aesthetic triangle of the breast, which is a new term and the key to a simplified shape analysis.

## Notes

### Acknowledgment

I thank Dr. Wolfgang Reimers for his support in the statistical analysis of the data. I also thank Dr. Holger Hofheinz for access to the 3D Vectra Imaging system at the Klinik am Rhein and the staff for putting up with me while being busy with the research study. I thank the Universitat Autonoma de Barcelona for educating me in breast surgery in the context of a Master degree course and for giving me the incentive to pursue research in this field.

### Ethical statement

All procedures performed in the study were done in accordance with the ethical standards of the institutional and national research committee and with the 1964 Helsinki declaration and its later amendments or comparable ethical standards.

### Competing interests

The author declares that she has no conflicting interests. This was an independent investigation into data obtained by the Vectra Camera System by the company Canfield Scientific Inc.

## Figures and Tables

**Table 1:**
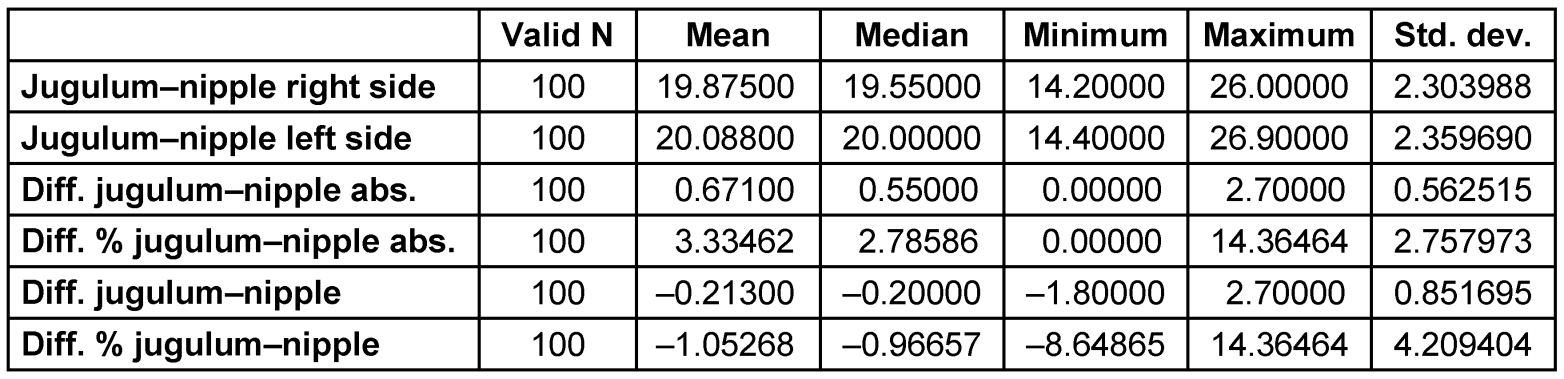
The distances between the jugulum and nipple on both sides of the breast

**Table 2:**
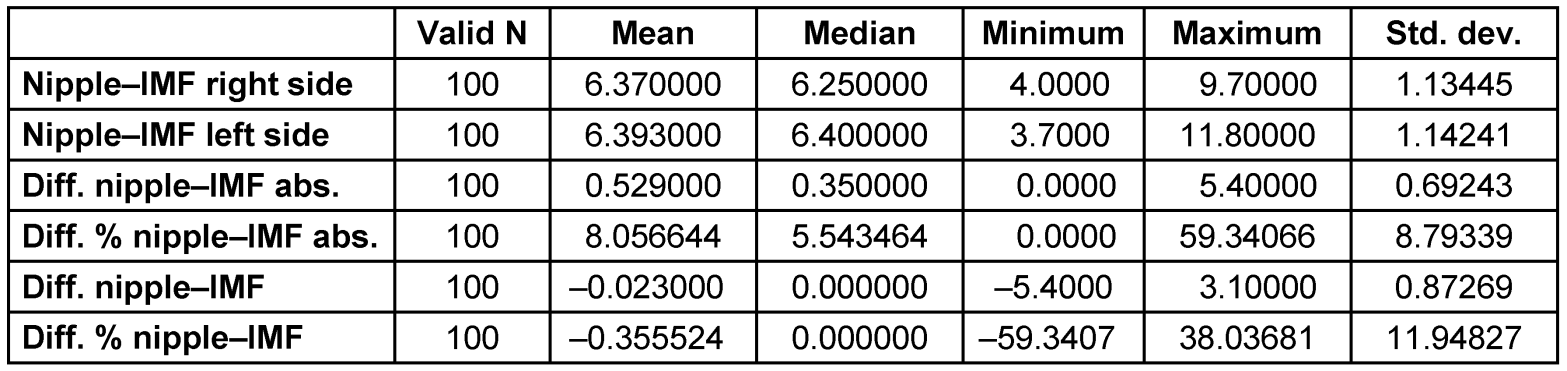
The distances between the nipple and inframammary fold (IMF) on both sides of the breast

**Table 3:**
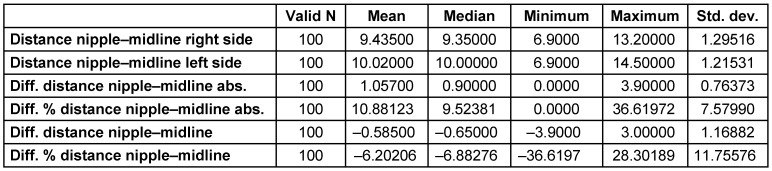
The distances between the nipple and midline on both sides of the breast

**Table 4:**
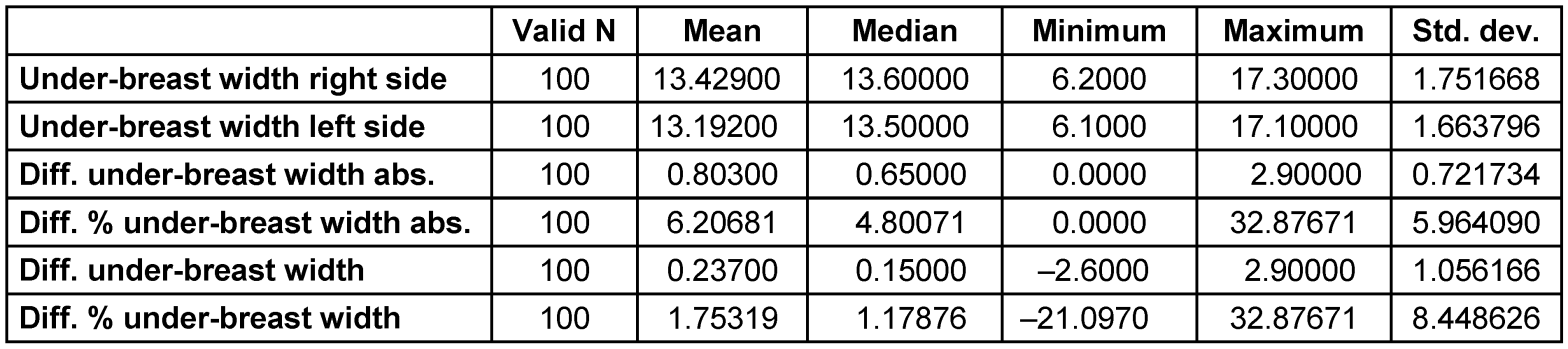
The under-breast width on both sides of the breast
